# Case Report: Fulminant Celiac Disease With Combination Immune Checkpoint Therapy

**DOI:** 10.3389/fimmu.2022.871452

**Published:** 2022-04-14

**Authors:** Ayo S. Falade, Kerry L. Reynolds, Leyre Zubiri, Vikram Deshpande, Florian J. Fintelmann, Michael Dougan, Meghan J. Mooradian

**Affiliations:** ^1^ Department of Medicine, Salem Hospital, Salem, MA, United States; ^2^ Department of Medicine, Massachusetts General Hospital, Boston, MA, United States; ^3^ Department of Pathology, Massachusetts General Hospital, Boston, MA, United States; ^4^ Department of Radiology, Massachusetts General Hospital, Boston, MA, United States

**Keywords:** immunotherapy, celiac disease, immune-related adverse effects, immune checkpoint inhibitors, immune-related celiac disease

## Abstract

Since the first approval of immune checkpoint inhibitors (ICIs) in 2011, these agents have rapidly become an integral treatment option across tumor types. However, with the increased adoption of ICIs, the incidence of immune-related adverse events (irAEs) continues to rise, and rare toxicity continues to be reported. Here, we present a case of a 70-year-old male patient with widespread metastatic melanoma who developed rapid onset anasarca and transaminitis after initiation of dual anti-PD-1/CTLA-4 inhibition with nivolumab and ipilimumab. An extensive workup was performed with serologies returning positive for anti-tissue transglutaminase immunoglobulin (tTG-IgA) and endoscopy revealing duodenal mucosal atrophy with duodenal biopsies confirming celiac disease. All symptoms resolved after initiation of a gluten-free diet without the addition of immunosuppression. This case highlights the importance of considering celiac disease in patients with suspected protein-losing enteropathy on ICI, the fulminant nature this uncommon irAE can present with, and underscores the broad differential clinicians must maintain when managing presumed irAEs.

## Introduction

Over the last decade, immune checkpoint inhibitors (ICIs) have revolutionized oncology care with these agents now approved in over a dozen tumor types with indications in the neoadjuvant, adjuvant, and metastatic setting. With their increasing use comes a critical need for clinicians to recognize the variable presentations of immune-related adverse events (irAEs). Luminal gastrointestinal (GI) irAEs are a well-known sequela of ICI (1) with common presentations ranging from mild dyspepsia to severe gastroenterocolitis; however, in rare cases, GI toxicity can also manifest as extraluminal symptoms (2).

ICI-associated celiac disease (ICI-CeD), though rare, is an established irAE (3–5). Similar to traditional celiac disease (CeD), a well-known T-cell-mediated reaction to dietary gluten, it frequently presents with diarrhea and vague abdominal discomfort. When a diagnosis is suspected in a patient with positive tissue transglutaminase (tTG)-IgA antibody, an upper endoscopy with a small bowel biopsy is recommended to confirm the diagnosis. Classic endoscopic and histologic features include mucosal inflammation, crypt hyperplasia, and villous atrophy of the small bowel (6). Recent data demonstrate that the clinical presentation, endoscopic findings, and response to gluten withdrawal in ICI-CeD mirror those seen in CeD (3). Whether ICI-CeD represents exacerbation of an underlying subclinical disease versus a *de-novo* condition is unclear. According to Badran et al., the approximate incidence of ICI-CeD based on a melanoma cohort is 0.3% of cases of diarrhea on ICI, which highlights the low incidence of CeD among patients treated with ICI therapy (3).

Here, we report an unusual and fulminant presentation of ICI-CeD in a patient with metastatic melanoma receiving combination nivolumab and ipilimumab.

## Case Report

**Figure 1:**
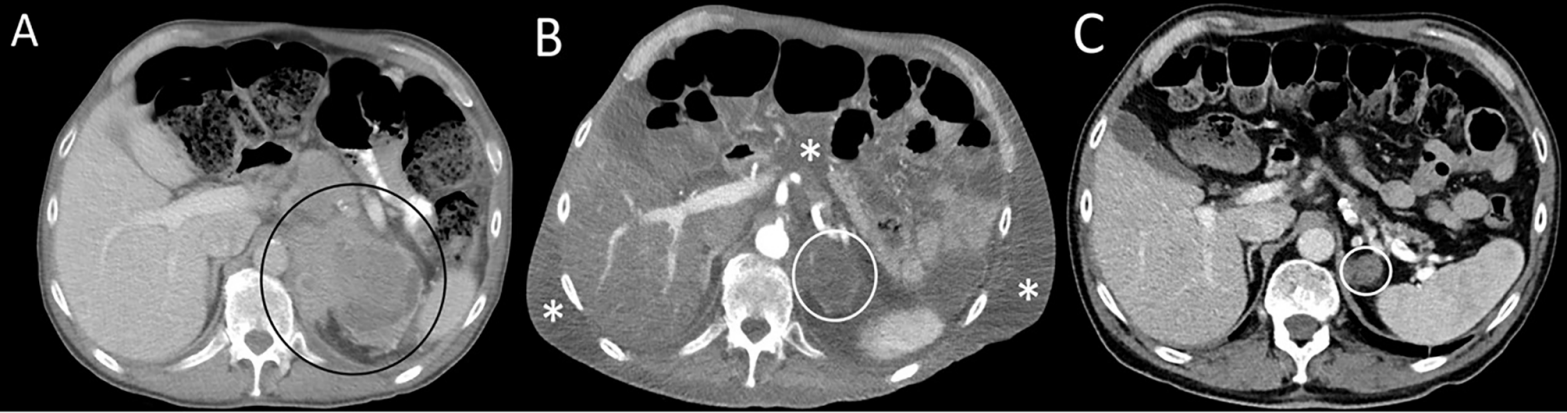
Serial axial computed contrast-enhanced computed tomography (CT) images of the upper abdomen demonstrate **(A)** a left adrenal mass (circle) 4 months before admission and prior to treatment, **(B)** decreased tumor size by more than 50% (circle) as well as new-onset ascites and soft tissue edema (asterisks) at the time of hospital admission following therapy with combination immune checkpoint inhibitor therapy; **(C)** resolution of ascites and soft tissue edema and continued tumor response (circle) 8 months after admission. The patient was not receiving therapy at the time of the follow-up CT.

**Figure 2:**
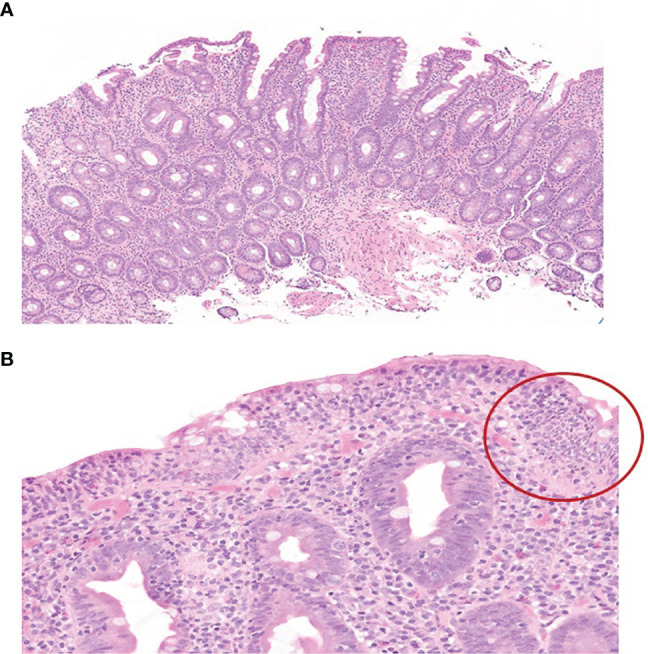
**(A)** H&E-stained slide (×10) of duodenal biopsy demonstrating marked villous blunting, crypt hyperplasia, and expansion of lamina propria by lymphocytes and plasma cells. **(B)** H&E-stained slide (×10) of duodenal biopsy highlights the marked intraepithelial lymphocytosis (circled) without evidence of epithelial injury or neutrophils.

A 70-year-old Caucasian male patient with widespread metastatic melanoma (**Figure 1A**) was initiated on first-line nivolumab (1 mg/kg) and ipilimumab (3 mg/kg) administered every 3 weeks. Restaging scans after two cycles of combination therapy demonstrated a partial response, and the patient reported an improvement in energy and appetite. However, shortly after cycle 2, he developed diarrhea, which slowly escalated from a baseline of two solid bowel movements per day to 4–6 per day by the end of cycle 4. These bowel movements were initially watery but became more formed over time, and he lacked additional GI complaints such as dyspepsia, nausea, vomiting, abdominal cramping, or hematochezia. Infectious stool studies, including *Clostridioides difficile* testing, were negative. Endoscopy was deferred given improvement in diarrhea. During this time, he developed 1+ edema of the bilateral lower extremities (right > left). A right lower extremity deep venous thrombosis was diagnosed, and he was started on low molecular weight heparin (LMWH). Despite LMWH, his edema worsened, and serial lab tests demonstrated a slowly downtrending albumin. Upon presentation to the clinic for consideration of cycle 5 of immunotherapy with single-agent nivolumab (his regimen consists of 4 cycles of ipilimumab and nivolumab followed by nivolumab monotherapy), he reported dyspnea on exertion and his exam revealed periorbital edema, ascites, and 3+ pitting edema of bilateral lower extremities. Laboratory evaluation demonstrated significant hypoalbuminemia (1.7 g/dl), hypophosphatemia (1.4 mg/dl), and a rising transaminitis (ALT 65 U/L and AST 62 U/L). Alkaline phosphatase (ALP) was 503 U/L, with the remaining liver function tests within normal limits. He was first admitted to an outside hospital where the cause of his symptoms could not be identified and subsequently transferred to a tertiary care facility for an expedited workup of a suspected immune-mediated toxicity. On arrival, he was hypotensive, and a broad differential was considered for his anasarca and hemodynamic compromise including sepsis, tumor progression, Budd–Chiari syndrome, nephritis with an evolving nephrotic syndrome, myocarditis, heart failure, endocrinopathies (including myxedema and adrenal insufficiency), and protein-losing enteropathy with potential concurrent hepatitis. An extensive workup was performed including viral hepatitis and CMV serologies and *Helicobacter pylori* stool antigen which were all negative. Urinalysis did not show proteinuria and the protein/urine creatinine ratio was normal. CT imaging of the chest, abdomen, and pelvis demonstrated continued tumor control although new bilateral ground-glass opacities (GGOs) and pleural effusions were noted, as well as diffuse soft tissue edema and ascites in the abdomen and pelvis (**Figure 1B**). These findings raised the concern for potential pulmonary and/or cardiac immune-mediated toxicities, as the occurrence of multiple irAEs is a common pattern in hospitalized patients, especially after treatment with combination therapy. A TTE demonstrated a normal ejection fraction. EKG and troponin levels were normal, with an NT-proBNP of 9,768. While hospitalized, his ALP continued to rise with a peak of 566 U/L with ALT/AST peaking at 91 and 71 U/L, respectively. Stool alpha 1-antitrypsin and tTG Ab IgA were sent. Gastroenterology was consulted due to concern for a protein-losing enteropathy, and an upper and a lower endoscopy were performed with extensive biopsies taken. Endoscopy visualized mild gastric erythema and diffuse mucosal atrophy throughout the duodenum with the visualized colon appearing normal. Duodenal biopsies demonstrated marked villous blunting, intraepithelial lymphocytosis, and expansion of lamina propria (**Figure 2**). Shortly after endoscopy was performed, stool anti-alpha 1-antitrypsin returned negative and tTG-IgA returned positive (>100 U/ml) confirming a diagnosis of celiac disease. In the hospital, the patient received intravenous albumin (12.5 g on October 6, 2020) and furosemide (10 mg IV on October 6, 2020, and 20 mg orally on October 8, 2020) which improved his hypotension, bilateral lower extremity edema, and dyspnea. He was ultimately initiated on a gluten-free diet once tTG-IgA resulted. The use of systemic immunosuppression was deferred. His edema resolved after several weeks of dietary adherence. Four months later (**Table 1**), labs demonstrated normalization of albumin values and a decline in tTG-IgA levels (4.40 U/ml). While recovering, immunotherapy was held. Subsequent CT scan (**Figure 1C**) demonstrated persistent disease control and resolution of volume overload.

**Table 1:** Lab values of interest on admission, discharge, and 3 months post-discharge.

	During admission (September 29, 2020)	On discharge (October 8, 2020)	3 months later (January 14, 2021)
Albumin (g/dl)	1.6	1.9	4.0
ALK phos (U/L)	460	371	68
ALT (U/L)	54	61	16
AST (U/L)	44	40	21
tTG-IgA (U/ml)	On October 1, 2020	On November 12, 2020	On March 5, 2021
	>100	16.88	4.40
Magnesium (mg/dl)	1.6	1.7	1.8
Calcium (mg/dl)	7.2	7.4	9.3
LDH (U/L)	237	217	162

## Discussion

Clinicians are now well trained to identify the most common irAEs. However, less frequent toxicities with atypical presentations remain challenging to diagnose. The initial differential diagnoses entertained in this case due to the severity of symptoms included the more common and/or well-known irAEs such as enterocolitis, pneumonitis, hepatitis, myocarditis, hypothyroidism, and adrenal insufficiency. However, after an extensive workup, this patient was found to have a fulminant presentation of ICI-CeD where the initial GI complaints were mild (grade 1 diarrhea) and largely resolved despite ongoing duodenal inflammation resulting in a significant protein-losing enteropathy and hepatitis.

Data on ICI-CeD are limited and largely comprised of case reports (4, 5, 7). The largest case series highlighted eight cases of ICI-CeD, confirmed by tTG-IgA. In this report, the authors compared ICI-CeD to CeD as well as to ICI-duodenitis. The clinical presentation and histological findings in CeD, ICI-CeD, and ICI-duodenitis were similar; however, patients with CeD and ICI-CeD had positive serologies, and specifically in cases of ICI-CeD, tTG-IgA titers ranged from 104 to >300 IU/ml. Notably, in our case, rather than the common presentation of mild upper GI complaints, ICI-CeD manifested as a severe clinical syndrome of malabsorption with significant hypoalbuminemia resulting in hypotension and diffuse anasarca. As seen in our case, effective management of ICI-CeD centers on strict adherence to a gluten-free diet rather than initiation of systemic immunosuppression. This dietary change does not onlylead to the resolution of symptoms but also spares patients from systemic immunosuppression, which may reduce ICI efficacy (8). In addition to emphasizing the optimal treatment of ICI-CeD and the importance of including this toxicity in the differential diagnosis of potential GI toxicity, this case highlights the extraluminal manifestations of ICI-CeD. Rather than a concomitant ICI hepatotoxicity, our patient’s transaminitis and elevated alkaline phosphatase were secondary to ICI-CeD. Though the exact mechanism of cryptogenic liver injury in celiac disease is unknown, levels often normalize after dietary gluten exclusion as was the case in our patient.

Like other irAEs, such as inflammatory arthritis or other rheumatologic disorders, it is difficult to discern if the development of ICI-CeD represents an unmasking of a long-standing subclinical disease or *de-novo* development of immune-mediated gluten sensitivity triggered by ICI use. In the absence of pretreatment tTG-IgA titers in this case, we are unable to discern the exact mechanism of this patient’s toxicity. Though ICI-CeD is rare, there may be utility to obtain tTG-IgA titers prior to ICI initiation to identify patients at risk for the evolution of subclinical disease to symptomatic gluten sensitivity. At this time, there are no reliable clinical factors (e.g., age, sex, tumor type, or type of ICI) that are associated with the development of ICI-CeD. Additionally, like other well-known immune-mediated toxicities, we currently lack predictive biomarkers (clinical and/or translational) to identify patients and/or tumor types at risk to develop ICI-CeD. Further research in this space is critical.

In conclusion, ICI-CeD is a rare irAE that often resembles ICI-mediated colitis; however, it can also present with extraluminal manifestations. As highlighted in our case, ICI-CeD can be a fulminant process. Clinicians needs to maintain a high level of suspicion for ICI-CeD in patients with new-onset GI complaints including LFT abnl as early and accurate diagnosis is important to avoid complications. Furthemore, with an accurate diagnosis of ICI-CeD, clinicians can employ the optimal treatment of gluten restriction rather than high-dose immunosuppresssion.

## Data Availability Statement

The original contributions presented in the study are included in the article/supplementary material. Further inquiries can be directed to the corresponding authors.

## Ethics Statement

Ethical review and approval was not required for the study on human participants in accordance with the local legislation and institutional requirements. The patients/participants provided their written informed consent to participate in this study. Written informed consent was obtained from the individual(s) for the publication of any potentially identifiable images or data included in this article.

## Author Contributions

AF and MM drafted and edited the case report. KR, LZ, and MD edited the case report. VD edited the case report and provided the pathology slides. FF edited the case report and provided and edited the images for publication. All authors contributed to the article and approved the submitted version.

## Conflict of Interest

MM served as a consultant or received honorarium from AstraZeneca, Bristol Myers Squibb, Immunia, Istari Oncology, and Nektar Therapeutics. MD received research funding from Novartis and Eli Lilly; received consulting fees from Tillotts Pharma, ORIC Pharmaceuticals, Partner Therapeutics, SQZ Biotech, AzurRx, Mallinckrodt Pharmaceuticals, and Moderna; received honoraria from WebMD, Experts at Your Fingertips, and Sandoz Academy; and is a member of the Scientific Advisory Board for Neoleukin Therapeutics. KR received research funding from Project DataSphere. LZ served as a consultant for Merck.

The remaining authors declare that the research was conducted in the absence of any commercial or financial relationships that could be construed as a potential conflict of interest.

## Publisher’s Note

All claims expressed in this article are solely those of the authors and do not necessarily represent those of their affiliated organizations, or those of the publisher, the editors and the reviewers. Any product that may be evaluated in this article, or claim that may be made by its manufacturer, is not guaranteed or endorsed by the publisher.
